# Nicotinic modulation of glutamate receptor function at nerve terminal level: a fine-tuning of synaptic signals

**DOI:** 10.3389/fphar.2015.00089

**Published:** 2015-04-29

**Authors:** Mario Marchi, Massimo Grilli, Anna M. Pittaluga

**Affiliations:** ^1^Department of Pharmacy, Pharmacology and Toxicology Section, University of Genoa, Genoa, Italy; ^2^Center of Excellence for Biomedical Research, University of Genoa, Genoa, Italy

**Keywords:** nicotinic receptors, AMPA receptors, NMDA receptors, synaptosomes, neurotransmitter release, receptor–receptor interactions, synaptic plasticity

## Abstract

This review focuses on a specific interaction occurring between the nicotinic cholinergic receptors (nAChRs) and the glutamatergic receptors (GluRs) at the nerve endings level. We have employed synaptosomes in superfusion and supplemented and integrated our findings with data obtained using techniques from molecular biology and immuno-cytochemistry, and the assessment of receptor trafficking. In particular, we characterize the following: (1) the direct and unequivocal localization of native α-amino-3-hydroxy-5-methyl-4-isoxazolepropionic acid (AMPA) and *N*-methyl-D-aspartate (NMDA) glutamatergic receptors on specific nerve terminals, (2) their pharmacological characterization and functional co-localization with nAChRs on the same nerve endings, and (3) the existence of synergistic or antagonistic interactions among them. Indeed, in the rat nucleus accumbens (NAc), the function of some AMPA and NMDA receptors present on the dopaminergic and glutamatergic nerve terminals can be regulated negatively or positively in response to a brief activation of nAChRs. This effect occurs rapidly and involves the trafficking of AMPA and NMDA receptors. The event takes place also at very low concentrations of nicotine and involves the activation of several nAChRs subtypes. This dynamic control by cholinergic nicotinic system of glutamatergic NMDA and AMPA receptors might therefore represent an important neuronal presynaptic adaptation associated with nicotine administration. The understanding of the role of these nicotine-induced functional changes might open new and interesting perspectives both in terms of explaining the mechanisms that underlie some of the effects of nicotine addiction and in the development of new drugs for smoking cessation.

## Introduction

Neuronal nicotinic receptors (nAChRs) are widely distributed in the rats’ central nervous system (CNS). They are located on different types of neurons, and are thought to serve different functions (Dajas-Bailador and Wonnacott, 2004). Accordingly, the outcome of the nAChRs stimulation depends on both the location of the cholinergic neurons and the specific type of neuron on which the receptors are expressed. Data from studies performed with genetically modified mice combined with neurotransmitter release and electrophysiological data consistently show that nAChRs exert a predominant role in the modulation of the release of neurotransmitters in the brain (Wonnacott, 1997).

Certainly, one of the most studied roles of nAChRs present on nerve endings is their ability to facilitate the release of several neurotransmitters (Arqueros et al., 1978; Wonnacott, 1997; Sher et al., 2004; Jones and Wonnacott, 2005; Marchi and Grilli, 2010). The effect of nicotine occurs throughout the activation of nAChR subtypes, which have different pharmacological and functional characteristics. One of the most abundant nAChRs present in the CNS, the α4β2 nAChR subtype, favors the entry of sodium and causes the depolarization that in turn activates the voltage operated calcium channels and thus facilitates the entry of calcium (Wonnacott, 1997; Dajas-Bailador and Wonnacott, 2004). Others, such as the α7 nAChR subtypes, are much more permeable to calcium (Bertrand et al., 1990) which when present in the cytoplasm-may activate the mobilization of calcium from intracellular stores (Dickinson et al., 2008). Throughout both of these mechanisms, nicotine evokes the release of several neurotransmitters (Turner, 2004; Zappettini et al., 2010). The EC_50_ value of nicotine falls within a micromolar range (McGehee and Role, 1995; Vibat et al., 1995; Buisson et al., 1996), while at concentrations equal to or less than 0.1 μM nicotine is really little or non-effective (Marchi et al., 2002; Zappettini et al., 2011).

However, quite interestingly, nicotine, may also modulate the functional responses of some receptors co-existing with nAChRs on the same nerve ending (Vizi and Lendvai, 1999; Sher et al., 2004; Parodi et al., 2006; Patti et al., 2006; Grilli et al., 2008, 2009a,b; Lin et al., 2010; Marchi and Grilli, 2010). This ability of nicotine in controlling receptor-mediated responses is relatively new and particularly intriguing when considering that sometimes it occurs also at nicotine concentrations insufficient to elicit neurotransmitter release. Briefly, the mechanism of receptor–receptor cross talk escapes that of control of neurotransmitter exocytosis but it finely tunes those mechanisms controlling in-out movements of receptors in synaptosomal plasma membranes. In particular, at the level of dopaminergic terminals, nicotine may exert a negative modulatory effect on the functionality of other receptors, coexisting with nAChRs, and this gives rise to a decrease of dopamine (DA) release if elicited by other neurotransmitter systems. Accordingly, nicotine used at low concentrations does not produce its typical stimulatory effect on DA release but conversely may cause inhibitory effects..

The modifications of receptor function sometimes are the outcome of a direct structural interaction between the two receptor systems involved, or are due to indirect effects mediated by a chain of events leading to this result. The most common mechanisms which underlie receptors’ modulation relate to changes in receptor affinity, variations in the ions permeability, and/or in the transduction signaling pathways, as well as changes in receptor trafficking. The interaction among co-expressed receptors may modulate neurotransmission function via several receptor systems (Raiteri et al., 1992; Pittaluga et al., 2000, 2005; Marchi and Grilli, 2010; Cong et al., 2011; Musante et al., 2011; Delille et al., 2013).

It is known that nAChRs coexist with glutamatergic α-amino-3-hydroxy-5-methyl-4-isoxazolepropionic acid (AMPA) and *N*-methyl-D-aspartate (NMDA) receptors on several neurons and particularly at nerve endings level (Marchi and Grilli, 2010; Marchi et al., 2014; Marks et al., 2014). The present review takes into account this new aspect of neuronal nicotinic activity with particular regard to the effects of nicotine on indirect modulation of glutamate and DA release. Of course, this particular effect of nicotine that occurs at the level of some presynaptic nerve terminals could even concern receptors present at the postsynaptic level thus providing a possible broader look at the action of nicotine in the brain.

## Isolated Nerve Terminals in Superfusion: An *in vitro* Model for Studying Functional Interaction between Presynaptic Receptors

Isolated nerve endings (synaptosomes) are subcellular particles of functionally active nerve tissue, which can be obtained easily by means of a special homogenization of brain tissues (Gray and Whittaker, 1962). These particles can be purified from the other components of the homogenate by centrifugation in a density gradient (Nagy and Delgado-Escueta, 1984; Dunkley et al., 2008). Synaptosomes have been characterized both morphologically and biochemically (Taupin et al., 1994; Feligioni et al., 2006). Morphologically, they resemble as small rounded pockets of 1–2 microns in diameter. They retain on the neuronal membrane structures present in the nerve ending *in vivo* (i.e., receptors, carriers, etc.). Therefore, when incubated in suitable physiological solutions, synaptosomes are capable of performing the neurochemical activities characteristic of the nerve terminals from which they originate. An important point is that synaptosomes are derived from neurons that were formed, developed, and differentiated in their natural environment, the living brain, allowing a reliable analysis of the events that occurs in CNS. More specifically, the synaptosomes’ neuronal origin as well as the lack of the neuronal/glial network that usually prime the physiological plasticity of neurons reduce eventual sources of bias for downstream analyses. This aspect must be taken into consideration when using other *in vitro* experimental models, such as primary neuronal cultures, which often have glial networks still present to improve culture conditions. The use of the superfusion technique in which synaptosomes are layered in a monolayer on microporous filters and up-down superfused with physiological solutions, has allowed us to overcome most of the problems associated with studies of neurotransmitter release with nerve endings in incubation (Raiteri et al., 1974). Under these experimental conditions, the superfusion medium removes the neurotransmitters released before they can interact and activate structures such as receptors or carriers present on the nerve endings, thereby excluding any possible indirect effects. Accordingly, under these experimental conditions the targets on the synaptosomal membranes such as receptors and/or carriers can be selectively activated by ligands present in the perfusion medium. Therefore, any effect on the release of a neurotransmitter may be attributed exclusively to an action on targets present on the nerve ending that specifically releases that neurotransmitter. In fact, neurotransmitter release can be triggered not only by depolarizing substances (KCl, veratrine, etc.), but also by drugs activating receptors present on the nerve terminals and which are stimulated by nicotinic and glutamatergic agonists. For all these reasons, this well-known preparation (Gray and Whittaker, 1962; Raiteri et al., 1974) remains an excellent model for studying molecular and subcellular mechanisms of neurotransmission.

Moreover, using synaptosomes in perfusion to study receptors that modulate the release of neurotransmitters, supplemented and integrated with data obtained by molecular biology, immuno-cytochemistry techniques, and by the assessment of receptor trafficking, enables us to obtain novel information. Indeed, we can provide relevant and specific information such as: (a) the direct and unequivocal localization of native receptors on the nerve terminal releasing the neurotransmitter under investigation; (b) the identification of the receptors function and the characterization of their transduction mechanisms; (c) the pharmacological characterization of these receptors and the mechanism of action of novel molecules acting on these sites [i.e., evaluation of the affinity and potency of agonist and antagonist drugs (EC_50_, IC_50_, pA_2_)]; and (d) the co-localization of different receptors on the same nerve endings and the presence of a synergistic or antagonistic interactions between them.

This approach can be also used for studying desensitization, trafficking of receptors, changes in their subunits structures, evaluation of positive or negative allosteric drugs, *ex vivo* assessment of receptor function after chronic treatment with drugs, changes in the environment or during development and aging (enriched environment, stress, etc.), and the neurotransmitters release from human tissue (Russo et al., 1993; Risso et al., 2004; Grilli et al., 2005, 2009a,b; Pittaluga et al., 2007; Musante et al., 2008; Summa et al., 2011; Marchi et al., 2012; Marrocco et al., 2014; Merega et al., 2014).

## Pre-Treatment with Nicotinic Agonists Modifies the Function of Glutamatergic NMDA Receptors Present on Nerve Terminals

The presence of the NMDA receptors (NMDARs) on neuronal membranes is quite important since it is connected to various plastic events of the synapse. Although NMDARs are not mobile like AMPARs, they also migrate from the endoplasmic reticulum to the membrane like all membrane proteins (Groc and Choquet, 2006; Pittaluga et al., 2007). It is therefore possible that they also undergo changes with regard to their presence at the level of neuronal membranes, and this may affect their function and modulatory activity. Several factors may trigger this receptor trafficking, including different stimuli derived by activation of receptors (Pittaluga et al., 2004, 2007; Summa et al., 2011). The specific interaction between the nicotinic cholinergic systems and the function of glutamatergic neurotransmission is a remarkable example.

**FIGURE 1:**
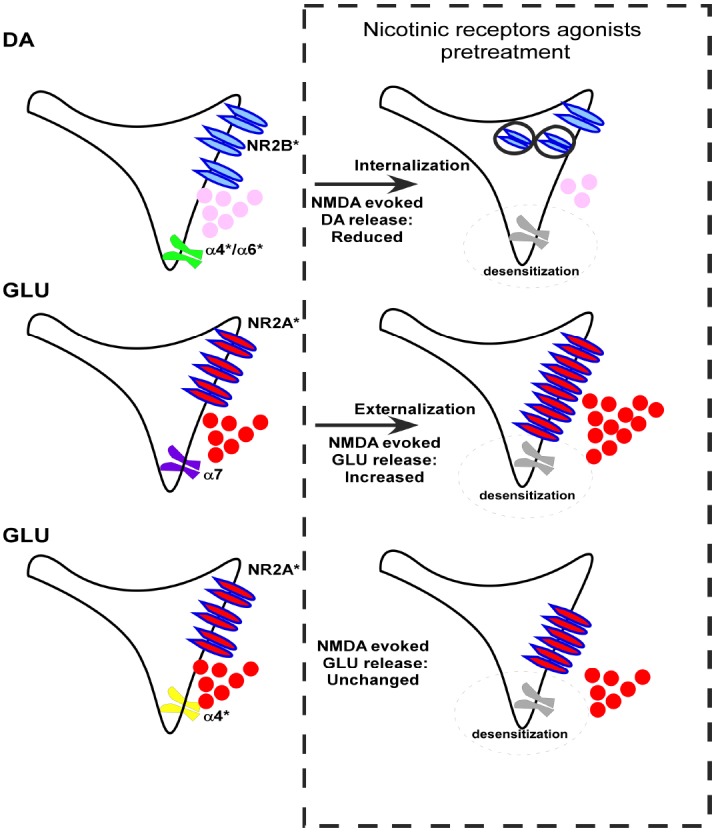
**A schematic representation of the consequences of the pre-treatment with nAChR agonists on the ability of NMDAR agonists (100 μM NMDA and 1 μM glycine) to evoke release of dopamine (DA) and glutamate (GLU) from rat nerve terminals and on the GluN2A and GluN2B NMDARs trafficking.** The asterisk indicates that the receptor subtype includes the presence of other different subunits in a variable manner in addition to the subunit specified.

A short pre-treatment of synaptosomes with nicotine (10 min) decreased the NMDA-induced DA release from nucleus accumbens (NAc) nerve terminals (Figure 1; Salamone et al., 2014). Since the nicotine pre-treatment failed to modify the 4-aminopyridine-induced DA overflow, changes in the exocytotic machinery of release do not account for nicotine-induced modifications of the NMDARs function (Salamone et al., 2014). These findings unequivocally demonstrate that both nAChRs and NMDARs are functionally located on the same nerve terminals where they also elicit calcium-dependent neurotransmitter release. Notably, results obtained by studying the calcium availability in individual nerve endings indicate that the pre-treatment with nicotine produced a significant decrease of the calcium transients evoked by the activation of NMDARs (Salamone et al., 2014).

Glutamate N2B subunits (GluN2B) are critical component of presynaptic NMDARs located on NAc DA nerve endings (Salamone et al., 2014). These subunits can undergo phosphorylation and their stoichiometry plays an essential role in the regulation of NMDAR channel properties (Akashi et al., 2009; Murphy et al., 2014), leaving open the possibility that the altered responses of NMDARs might involve either a rearrangement of subunits assembly or a modification of the phosphorylation of NMDARs. Alternatively, a change in NMDARs trafficking typified by a reduction of the number of NMDARs present at the plasma membrane level of DA nerve endings may also occur. Interestingly, this latter hypothesis has been clearly favored since biotinylation studies unveiled a reduced density of GluN2B subunits in NAc synaptosomal plasma membranes following nicotine pre-treatment (Salamone et al., 2014). Altogether, these findings demonstrate a functional interaction, within the same individual dopaminergic terminal, between nAChR and NR2B-containing NMDARs, through an ability of nicotinic agents to induce endocytosis of these latter receptors (Figure 1; Salamone et al., 2014).

With regard to the possible role of specific subtypes of nAChR involved in this mechanism, we must remember that different nAChR subtypes including α-conotoxin-sensitive (α6β2β3, α4α6β2β3, α6β2, and α4α6β2) and α-conotoxin- insensitive (α4β2 and α4α5β2) subtypes mediate nicotine-evoked DA release from striatal synaptosomes (Kaiser et al., 1998; Salminen et al., 2007). Since the inhibitory effect of nicotine pre-treatment was partially counteracted by α conotoxin MII, it is likely that nAChRs containing the α6 subunit, which is abundant in the NAc (Exley et al., 2008; Hendrickson and Tapper, 2014, and references therein) are also involved. The pre-treatment (10 min) with 5IA85380, a α4/α6 nicotine agonist, further confirms this hypothesis. Interestingly, preclinical evidence supports the importance of mesolimbic α6 containing nAChRs in the nicotine addiction (Exley et al., 2008; Brunzell, 2012). Conversely, α7-nAChR subtypes do not appear to be involved in any kind of interaction with glutamatergic receptors in NAc DA terminals as heralded also by the absence of effects after the pre-treatment (10 min) with choline (Salamone et al., 2014).

However, in other neurotransmitter systems it seems that even α7-nAChRs might be implicated in a cross-talk between nicotinic and glutamatergic receptors. A recent report focusing on glutamatergic synapses indicated that α7-nAChRs might enhance the presynaptic surface expression of NMDARs leading to an increased glutamate release during early synaptic development (Lin et al., 2010). Interestingly, the NMDARs modulating glutamate release are apparently different from those modulating the release of other neurotransmitters in several brain areas (Pittaluga et al., 2001). It can be therefore suggested that the interaction between nAChRs and NMDARs could be differently organized in different types of nerve terminals, possibly depending on the different nAChRs and NMDARs subtypes involved. In support of this hypothesis, a pre-treatment (10 min) of NAc glutamatergic synaptosomes with nicotine or the α 7 agonists choline caused a significant increase of the NMDA-evoked overflow of [^3^H] D-ASP while, conversely, the pre-treatment (10 min) of glutamatergic nerve endings with the α4/α6 nicotinic agonist 5IA85380 was ineffective (Figure 1; Zappettini et al., 2014).

The possibility that nicotinic and NMDA receptors coexist and may interact on glutamatergic terminals is not surprising. Indeed, the presence of α4β2 and α7 AChRs on glutamatergic nerve endings is well documented in several brain regions (for a review, see Vizi and Lendvai, 1999) as well as the presence of NMDA receptors that modulate the release of glutamate (Luccini et al., 2007; Zappettini et al., 2010; Musante et al., 2011). The pharmacological characterization indicates that GluN2A subunits participate in the expression of these presynaptic NMDARs located on glutamatergic nerve endings, which therefore differ from those containing GluN2B subunits present on NAc DA nerve terminals (Zappettini et al., 2014). Thus, at least some of these NMDARs are functionally expressed and co-localized both with α7 nAChRs with whom they can functionally interact. Furthermore, the pre-treatment with nicotine or choline produced a significant increase of the calcium transients evoked by the activation of NMDARs; this event occurred only in glutamatergic nerve endings (Zappettini et al., 2014). This finding is therefore consistent with the data summarized above, and supports the hypothesis that the facilitation of the NMDA-evoked overflow could be due to an increase of the synaptosomal calcium transients (Zappettini et al., 2014), yet considering that calcium influx can influence directly neurotransmitter release. It is also important to recall that NMDARs coexist also with α4β2 nAChR subtypes in glutamatergic terminals, but in this case without any functional interaction among them (Figure 1; Zappettini et al., 2014). Interestingly, biotinylation studies have shown an increased density of GluN2A subunits in synaptosomal plasma membranes following nicotine and choline pre-treatment but no changes after preincubation with 5IA85380 (Zappettini et al., 2014). These findings demonstrate the ability of α7 nicotinic but not of α4β2 agonists, to induce an increase in the trafficking of GluN2A-containing NMDARs and consequently an increase of the functional responses.

In conclusion, the nicotine-evoked internalization of NMDARs present on DA nerve endings appears not to depend upon the activation of one particular subtype of nAChR, but rather on the characteristics of the NMDARs coexisting with nAChRs on the same nerve terminal. Conversely, the increase of the response of the NMDARs present on the glutamatergic nerve endings after nicotine pre-treatment might depend on two concomitant factors: (a) the activation of a specific nAChR subtype (α7), and (b) the coexistence of these receptors with NMDARs which contain the GluN2A subunit (Figure 1; Salamone et al., 2014; Zappettini et al., 2014).

## Pre-Treatment with Nicotinic Agonists Modifies the Function of Presynaptic Glutamatergic AMPA Receptors

**FIGURE 2:**
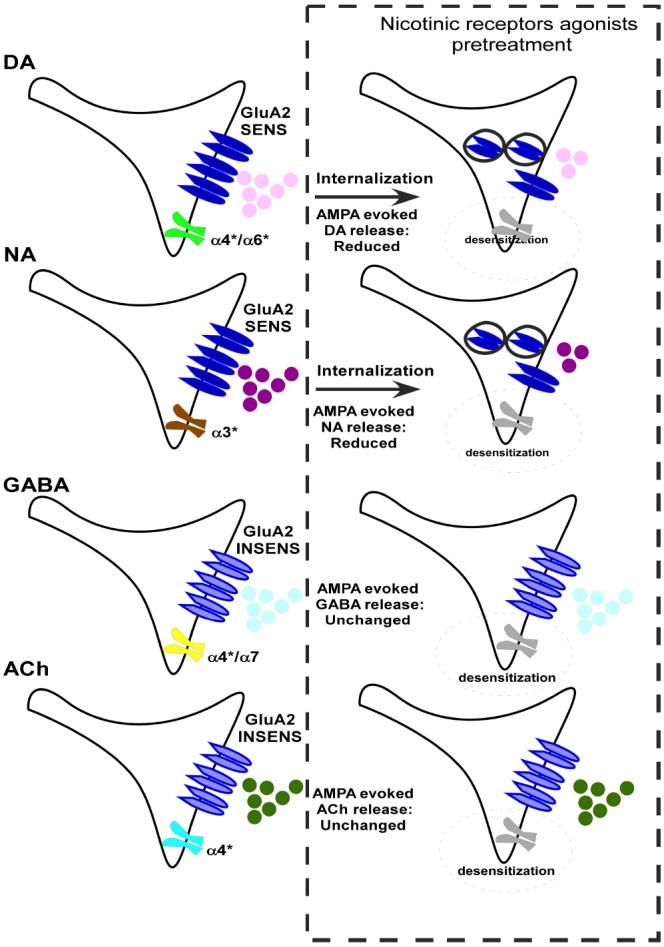
**A schematic representation of the consequences of the pre-treatment with nAChR agonists on the ability of NMDAR agonists (100 μM NMDA and 1 μM glycine) to evoke release of dopamine (DA) and noradrenaline (NA), acetylcholine (ACh) and GABA from rat nerve terminals and on the GluA2-cyclothiazide sensitive (GluA2 SENS) and GluA2-cyclothiazide insensitive (GluA2 INSENS) AMPARs trafficking.** The asterisk indicates that the receptor subtype includes the presence of other different subunits in a variable manner in addition to the subunit specified.

Alpha-amino-3-hydroxy-5-methyl-4-isoxazolepropionic acid are largely responsible for the rapid post-synaptic response to glutamate, are widely present in the CNS, and are differently distributed both at the post- and presynaptic level. Accordingly, a significant percentage of dopaminergic terminals present in the NAc possess GluA2 receptor subunits as demonstrated by functional and immuno-cytochemistry studies (Grilli et al., 2012). The AMPARs are highly dynamic in their distribution and can cycle rapidly among the synaptic membrane and intracellular compartments (Henley, 2003; Pittaluga et al., 2006). As a confirmation of this mobility, even within the nerve endings, the pre-treatment (10 min) of NAc synaptosomes with nicotine caused a significant reduction of the AMPA-evoked overflow of DA from NAc nerve terminals. Accordingly, the loss of function of AMPARs in nicotine pre-treated synaptosomes was accompanied by a decreased insertion of GluA2 subunit proteins in the presynaptic component of synaptosomal plasma membranes in the nicotine-treated nerve endings (Figure 2; Grilli et al., 2012). Interestingly, the decrease of the AMPA-induced DA overflow in nicotine-pre-treated NAc dopaminergic terminals was significantly counteracted in synaptosomes containing entrapped pep2-SVKI, a peptide known to compete for the binding of GluA2 subunit to scaffolding proteins involved in AMPARs internalization. Thus, it is likely that nicotine pre-treatment could favor the internalization of GluA2-containing AMPARs located on dopaminergic NAc terminals. This event, however, seems to occur in a selected population of nerve terminals, since AMPA-induced releasing effect was halved, but not abolished, by nicotine (Figure 2; Grilli et al., 2012).

Interestingly, the nicotine-induced changes to release-regulating presynaptic AMPARs was not limited to DA terminals, but could also be observed when studying the AMPA-evoked release of noradrenaline from hippocampal nerve terminals (Grilli et al., 2012). However, nicotine-induced adaptation of AMPARs does not represent a generalized event that occurs in all nerve endings. Indeed, the pre-treatment of NAc synaptosomes to nicotine failed to cause significant changes of the AMPA-evoked overflow of γ-aminobutyric acid (GABA) and ACh from these terminals (Figure 2; Grilli et al., 2012). The most likely explanation for the selective endocytosis of AMPARs that modulates DA and NA release can be the presence of similar cyclothiazide-sensitive AMPA receptors endowed with GluA2 subunits, which are susceptible to the internalization (Pittaluga et al., 1999, 2006; Ghersi et al., 2003; Grilli et al., 2012). Conversely, the AMPA receptors controlling acetylcholine and GABA release are cyclothiazide-insensitive and did not undergo a GRIP/ABP/PICK1-dependent constitutive trafficking (Pittaluga et al., 1997, 2006).

Due to the very heterogeneous presence of nAChRs on dopaminergic and noradrenergic nerve endings (Sershen et al., 1997; Zoli et al., 2002; Kennett et al., 2012; Pistillo et al., 2015, and references therein), it is very difficult to correlate specific nAChR subtypes with the internalization of AMPA receptors. In any case, the involvement of α7 nAChR subtype can be excluded based on the lack of effect of the specific α7 agonist choline due to the absence of this nAChR subtype on both dopaminergic and noradrenergic terminals (Clarke and Reuben, 1996; Kaiser and Wonnacott, 2000).

## Conclusions

As summarized above, studies have shown that in the rat NAc the function of specific GluRs present on the dopaminergic and glutamatergic nerve terminals can be negatively or positively regulated in response to a brief activation of nAChRs (Grilli et al., 2012; Salamone et al., 2014; Zappettini et al., 2014). The effect occurs rapidly and seems to be entirely caused by the trafficking of AMPA and NMDA receptors. However, this takes place only in certain nerve terminals and after the activation of either α7 or non-α7 nAChR subtypes. It is quite important to note that these effects occur at very low concentrations of nicotine (Salamone et al., 2014). Interestingly, these nicotine levels, which alone are unable to evoke release of neurotransmitters, are compatible with the nicotine concentrations present in the venous plasma of heavy smokers (Benowitz, 1988; for a review, see Matta et al., 2007) or even of people under nicotine replacement therapy for smoking cessation.

Moreover, it is important to note that nicotine at these concentrations switches its typical stimulatory effects on DA release into an inhibitory effect; this phenomenon could be a fine-tuning of synaptic signals in response to the increased glutamatergic activity mediated by the upregulation of NR2A-containing NMDARs.

The dynamic control by cholinergic nicotinic system of glutamatergic NMDA and AMPA receptors might therefore represent an important neuronal adaptation associated with nicotine administration. If and how this event may have a functional relevance or may play an important role in the clarification of the mechanism of action of nicotine may be worth investigating in the future. Indeed, the understanding of the role of these nicotine-induced functional changes might open new and interesting perspectives in terms of explaining the mechanisms that underlie some of the effects of nicotine addiction and the development of new drugs for smoking cessation.

It is rather complicated to contextualize the interaction of these two neurotransmitter systems mainly because of two reasons: (1) many neurons are able to interact to each other without making synapses. Recently, functional and morphological evidence has clearly demonstrated that most neurotransmitters can diffuse once released and achieve distant targets in the CNS, provided that the target neurons are equipped with receptors (Lendvai and Vizi, 2008); and (2) nACh, AMPA and NMDA receptors are located both synaptically and extrasynaptically (Groc et al., 2009, and references therein; Petralia et al., 2009). In fact, it has been proposed that GluNR2B-NMDARs are mostly extrasynaptically while GluNR2A-NMDARs seem to be located mostly at synaptic level (Groc et al., 2009, and references therein, Petralia et al., 2009; Sanz-Clemente et al., 2013). Therefore, further investigation on the interaction of these two neurotransmitter systems at the nerve terminal level will hopefully shed light on the role that this mechanism could play both in normal physiology as well as in nicotine dependence. Of relevance, Pistillo et al. (2015) reviewed the molecular, functional, and behavioral mechanisms involved in the nicotine-induced effects in the mesocorticolimbic system, and discussed the dopaminergic and glutamatergic circuits within these CNS regions.

The cellular and molecular mechanisms that regulate the presence of NMDA and AMPA receptors on neurons in the CNS have been thoroughly investigated over the last decades. There are several identified mechanisms that can explain the GluRs trafficking and the consequent impact that these events might have on the receptors responses (Zhou et al., 2015). NMDAR surface trafficking is subunit dependent being GluN2A-NMDARs less mobile and more retained within synapse than GluN2B-NMDARs. Approximately 30–40% of surface GluN2B-NMDAR are mobile and the average of synaptic residency time appears to be minutes (Tovar and Westbrook, 2002; Bard and Groc, 2011, and references therein).

It has been recently reported that NMDARs may also diffuse on the surface of neurons in a highly dynamic process (Groc et al., 2009). The mobility of presynaptic receptors on the neuron surface might therefore generate different functional responses (Gomez-Varela and Berg, 2013). How and through the activation of which mechanisms nicotine could control the surface dynamics of GluRs at the nerve terminal levels is at present not easy to explain. However, although we cannot establish whether the pre-treatment with nicotine is able to influence the rapid and lateral redistribution of GluRs at the membrane level, we can reasonable assume that the changes in the functional response of GluRs might to be linked to their trafficking from the cytoplasm to the surface of the terminal. Additional investigation is needed to clarify which mechanism(s) regulating the trafficking of AMPA and NMDA receptors so far reported is/are triggered or inhibited by the pre-treatment with nicotine. In fact, a physical interaction between α7 nAChR and the GluNR2A- NMDAR has been recently reported (Li et al., 2013). Therefore, the possibility that the pre-treatment with α7 agonists might upregulate the α7 nAChR/NMDAR complex and consequently enhance GluNR2A-NMDARs cell surface expression has to be also considered.

Several lines of evidence in laboratory animals and preclinical studies demonstrate that different glutamatergic receptors are critically involved in several nicotine-mediated effects (Vizi and Lendvai, 1999; Laviolette and Van Der Kooy, 2004; Dani and Bertrand, 2007; Markou, 2008; Timofeeva and Levin, 2011; Zhong et al., 2014). This dynamic control by cholinergic nicotinic system of both NMDA and AMPA receptors might therefore be crucial for many forms of synaptic plasticity including events linked to nicotine dependence. Moreover, this event should be also relevant to understand the interplay between nAChRs and NMDARs in neurodegenerative diseases. Epidemiological studies have identified a negative correlation between smoking and the development of neurodegenerative disorders such as Parkinson’s disease, and in some studies, Alzheimer’s disease (Picciotto and Zoli, 2008). These findings have been attributed to the ability of nicotine to act as a neuroprotective agent. Indeed, a certain number of studies demonstrated that nicotine can protect against neuronal death *in vitro* and *in vivo* (Dajas-Bailador et al., 2000; Jonnala and Buccafusco, 2001; Quik et al., 2007; Picciotto and Zoli, 2008; Shimohama, 2009; Akaike et al., 2010; Bordia et al., 2015). However, it is also very important to recall that several studies have reported that nicotine is neurotoxic to several neuronal subtypes, including immature neurons (Trauth et al., 2000; Laudenbach et al., 2002).

Although relevant, our findings are insufficient to support a strict correlation among these new nicotine-induced plastic modifications at nerve terminals, and the events that underlie nicotine addiction as well as nicotine-induced promnesic and neuroprotectant actions. Nevertheless, the working hypothesis is promising and surely deserves future investigation aimed at highlighting the roles that nicotine /glutamate receptor functional cross talk play in the mechanism of synaptic plasticity induced by nicotine.

### Conflict of Interest Statement

The authors declare that the research was conducted in the absence of any commercial or financial relationships that could be construed as a potential conflict of interest.

## Abbreviations

AMPA: α-amino-3-hydroxy-5-methyl-4-isoxazolepropionic acid
CNS: central nervous system
DA: dopamine
GABA: γ-aminobutyric acid
GluN2B/GluN2A: Glutamate N2B/N2A subunits
NAc: nucleus accumbens
nAChR: nicotinic acetylcholine receptor
NMDA: *N*-methyl-D-aspartate
NMDARs: NMDA receptors.
